# Influence of a patient transfer network of US inpatient facilities on the incidence of nosocomial infections

**DOI:** 10.1038/s41598-017-02245-7

**Published:** 2017-06-07

**Authors:** Juan Fernández-Gracia, Jukka-Pekka Onnela, Michael L. Barnett, Víctor M. Eguíluz, Nicholas A. Christakis

**Affiliations:** 1 Harvard T.H. Chan School of Public Health, 677 Huntington Ave, Boston, MA 02115 USA; 2 Institute for Cross-Disciplinary Physics and Complex Systems, Campus Universitat de les Illes Balears, Carretera de Valldemossa, km 7,5 Edificio Científico-Técnico, 07122 Palma de Mallorca, Islas Baleares Spain; 3 Department of Medicine, Department of Sociology, and Yale Institute for Network Science, Yale University, P.O. Box 208263, New Haven, CT 06520-8263 USA

## Abstract

Antibiotic-resistant bacterial infections are a substantial source of morbidity and mortality and have a common reservoir in inpatient settings. Transferring patients between facilities could be a mechanism for the spread of these infections. We wanted to assess whether a network of hospitals, linked by inpatient transfers, contributes to the spread of nosocomial infections and investigate how network structure may be leveraged to design efficient surveillance systems. We construct a network defined by the transfer of Medicare patients across US inpatient facilities using a 100% sample of inpatient discharge claims from 2006–2007. We show the association between network structure and *C. difficile* incidence, with a 1% increase in a facility’s *C. difficile* incidence being associated with a 0.53% increase in *C. difficile* incidence of neighboring facilities. Finally, we used network science methods to determine the facilities to monitor to maximize surveillance efficiency. An optimal surveillance strategy for selecting “sensor” hospitals, based on their network position, detects 80% of the *C. difficile* infections using only 2% of hospitals as sensors. Selecting a small fraction of facilities as “sensors” could be a cost-effective mechanism to monitor emerging nosocomial infections.

## Introduction

Healthcare-associated infections are a significant source of morbidity and mortality, imposing substantial clinical and financial costs to the US health care system^1–6^. Many infections have a common reservoir in inpatient settings such as hospitals and rehabilitation facilities, but they are difficult to monitor on a national scale. A 2013 Centers for Disease Control and Prevention (CDC) report on antibiotic-resistant bacteria identified the lack of infrastructure to detect and respond to emerging resistant infections as a pressing gap^2^. While patient transfers could plausibly act as a mechanism for epidemiologic spread from facility to facility, only a few studies have investigated the possible role of transfers for the spread of infections at the country level, which constitutes arguably the biggest scale for these kind of systems. Some studies have focused on the structure of the nationwide critical care transfer network^7–10^, while others have had a more restricted scope, limited to geographical units such as counties or states^11–14^. In this study, we consider nationwide transfers of Medicare patients 65 or older, who constitute about 15% of the US population^15^, and about 37% of all hospital admissions^16^. This population is also arguably at highest risk for morbidity and mortality from health care associated infections.

As a case study of nosocomial infections we use data on *Clostridium difficile [C. difficile]*, which is an anaerobic, gram-positive, spore-forming bacteria that occurs frequently in health care settings. It is found in >20% of patients who have been hospitalized for more than one week. The disease is spread by ingestion of *C. difficile* spores, which are very hardy and can persist on environmental surfaces for months without proper hygiene^17^. *C. difficile* associated infections reached half a million in the United States only, with 29,000 patients deaths, 15,000 of which were estimated to be directly caused by C. difficile infections (80% of patients 65 or older). Furthermore, approximately two thirds of the C. difficile infections are associated with a stay in an inpatient facility^18^.

To better understand the potential role of Medicare patient transfers in the spread of health care associated infections, we pursue three interconnected aims. First, we investigate the structure of the facility-to-facility Medicare patient transfer network in the US. Second, we correlate the incidence of nosocomial infections (using *Clostridum difficile [C. difficile]* as a case study) on a national scale with properties of this network. Note, however, that in the absence of genetic data to ascertain if the bacterial strains coincide, complete certainty on the routes of dispersal of the pathogens is not possible. And third, we develop and propose a scalable network based method for monitoring the system through “sensor” hospitals against the spread of these infections.

## Results

### Study population

The average age in the study population was 77.3 years, with 55.8% female patients, and 85% white. As expected, the cohort had high rates of chronic illness (Table S1).

### Properties of the transfer network

**Figure 1:**
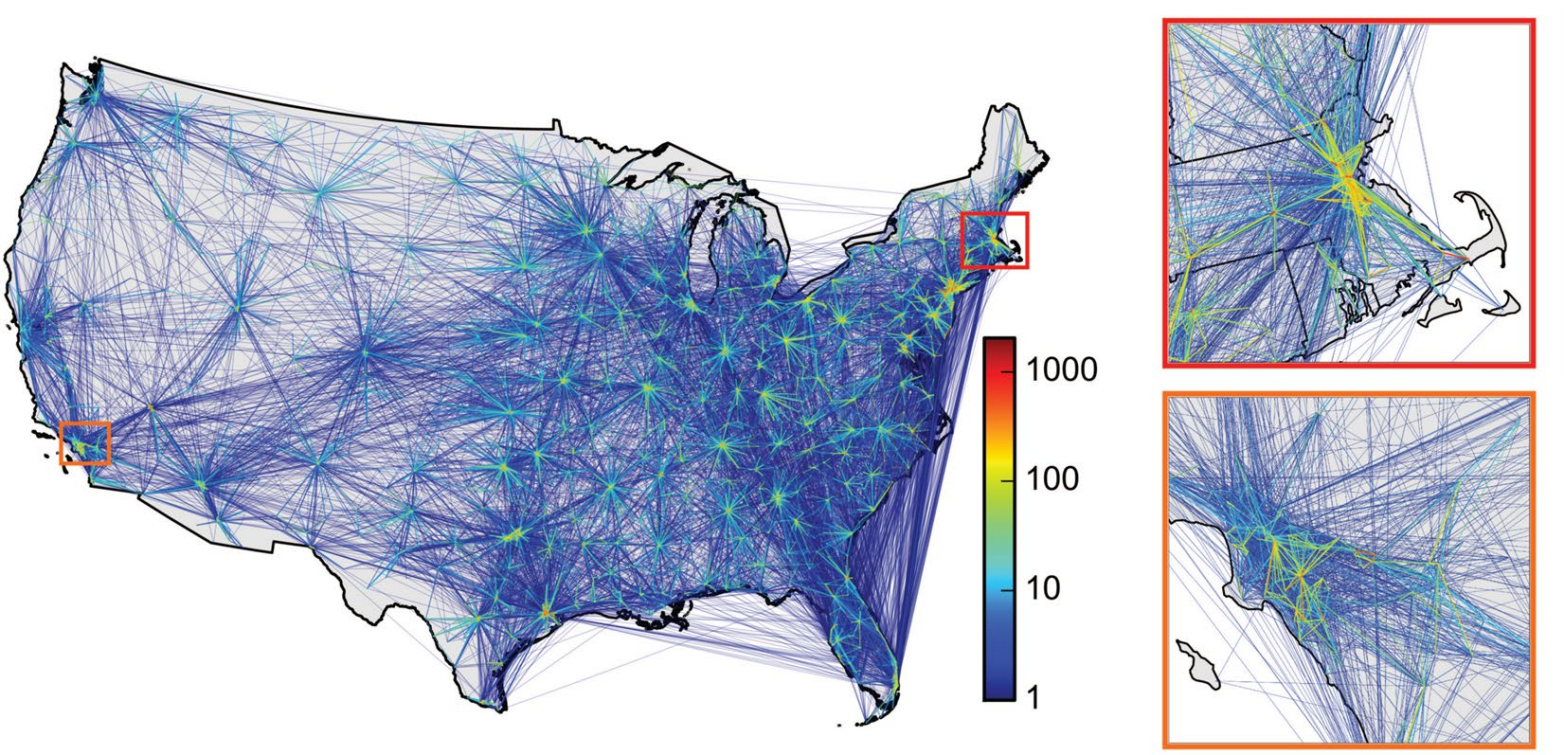
Facility transfer network. The network consists of facilities that are connected by daily transfers of patients, here aggregated over the two-year period. Edge color encodes the number of patients transferred through each connection. The insets show this network around Boston (upper inset) and around Los Angeles (lower inset). The maps were created using the Basemap Matplotlib Toolkit 1.0.8 (http://matplotlib.org/basemap/) for Python^24^.

The transfer network showed strong seasonal, monthly, and weekly cycles of patient transfers. The topology of the network and geography of patient transfers were closely related, with 90% of transfers between facilities less than 200 km apart. On average, over the 2-year period, a facility sent patients to 13.55 ± 0.15 (SE) other facilities and received patients from 13.55 ± 0.25 other facilities. (The two means necessarily coincide in a directed network because each directed edge has an outgoing end and an incoming end). The average number of patients transferred per edge in the 2-year period was 12.3 ± 0.63 (SE). A representation of the aggregated network is shown in Fig. 1 (See SM for more details).

Examining network characteristics by facility type, on average, general hospitals received transfers from 12.9 other institutions and sent transfers on average to 15.1 other facilities. In contrast, rehabilitation facilities on average received transfers from 24.7 other institutions and sent transfers to 9.4 other facilities (Table 1).

**Table 1:** Network characteristics by type of hospital.

Hospital Type	Acute general medical-surgical	Rehabilitation facilities	Other facilities
Number of hospitals	4546	526	535
Number of beds (Mean (SD))	165 (180)	70 (108)	134 (162)
**Network Measures (Mean (SD))**			
In-degree	12.9 (19.7)	24.7 (14.9)	8.4 (14.2)
In-strength	120 (265)	693 (675)	50 (156)
Out-degree	15.1 (11.4)	9.4 (6.3)	4.9 (7.5)
Out-strength	191 (266)	89.2 (84.8)	39 (184)

In-degree refers to the number of hospitals from which a hospital receives transferred patients. In-strength is the total number of transferred patients a hospital receives. Out-degree is the number of hospital to which a hospital transfers patients, while out-strength is how many patients a hospital transfers to other hospitals.

### Spread of *C. difficile* infections

**Figure 2:**
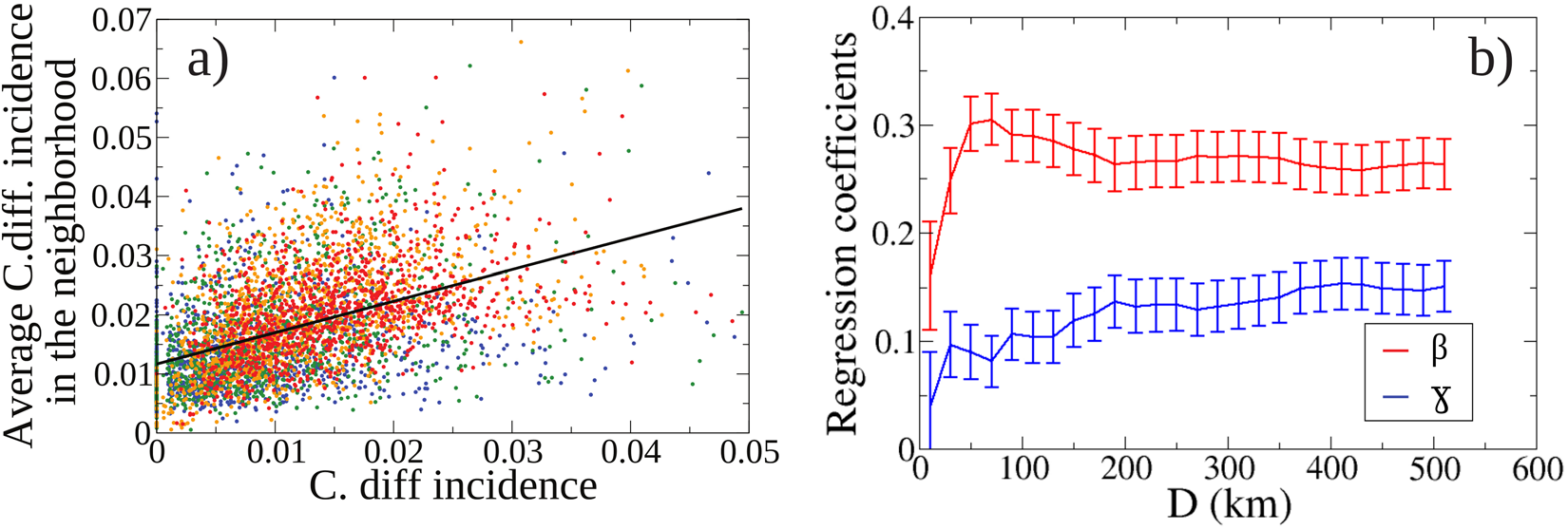
*C. difficile* incidence in a general facility and its neighbors. (**a**) Here we plot the *C. difficile* incidence of every general facility with less than 0.05 incidence on the x-axis and the average *C. difficile* incidence of the neighboring facility in the transfer network, where a neighboring facility is one that either sends it patients to or receives its patients from the case facility. Facility size in beds is represented by color: the first (lowest) quartile (in size) of facilities shown is plotted in blue, the second in green, third in orange and the fourth (largest) quartile in red. 74 hospitals (1.6% of sample) with *C. difficile* incidence greater than 0.05 were excluded from the plotting area. (**b**) Logistic regression coefficients as a function of distance. Coefficient for the average incidence in network neighbors (red) and not linked through transfers (blue) up to a distance *D*. Error bars show the 95% CI. This result can be interpreted in terms of z-scores: at a distance of 100 km, an increase of one standard deviation in the average *C. difficile* incidence in hospitals connected through the transfer network was associated with an average increase of 33.6% in the odds of a patient being diagnosed *C. difficile*, while for non-connected hospitals the increase was only of 10.5%.

We examined the relationship between *C. difficile* incidence for each facility and the average *C. difficile* incidence for its network neighbors (Fig. 2). As shown in Fig. 2a, these parameters were significantly correlated (Pearson correlation coefficient 0.48, 95% CI: 0.46, 0.50). Using linear regression, a 1% change in the *C. difficile* incidence of facility was associated with a change of 0.53 ± 0.02% in the average *C. difficile* incidence of network neighbor facilities. (p < 0.001, see SM for subgroup analysis by facility type and adjusting for hospital size, which shows only a marginal effect of hospital size).

We further examined the strength of the association in *C. difficile* incidence of hospitals linked by transfers up to a certain distance *D* versus other non-connected nearby hospitals. Across all transfer distances examined, there was a stronger association between a hospitals’ *C. difficile* incidence with its linked neighbors than with non-connected hospitals (Fig. 2b). For example, at a distance of 100 km, an increase of one standard deviation in the average *C. difficile* incidence in hospitals connected through the transfer network was associated with an average increase of 33.6% in the odds of a patient being diagnosed *C. difficile*, while for non-connected hospitals the increase was only of 10.5%.

### Monitoring the system for hypothetical epidemics

**Figure 3:**
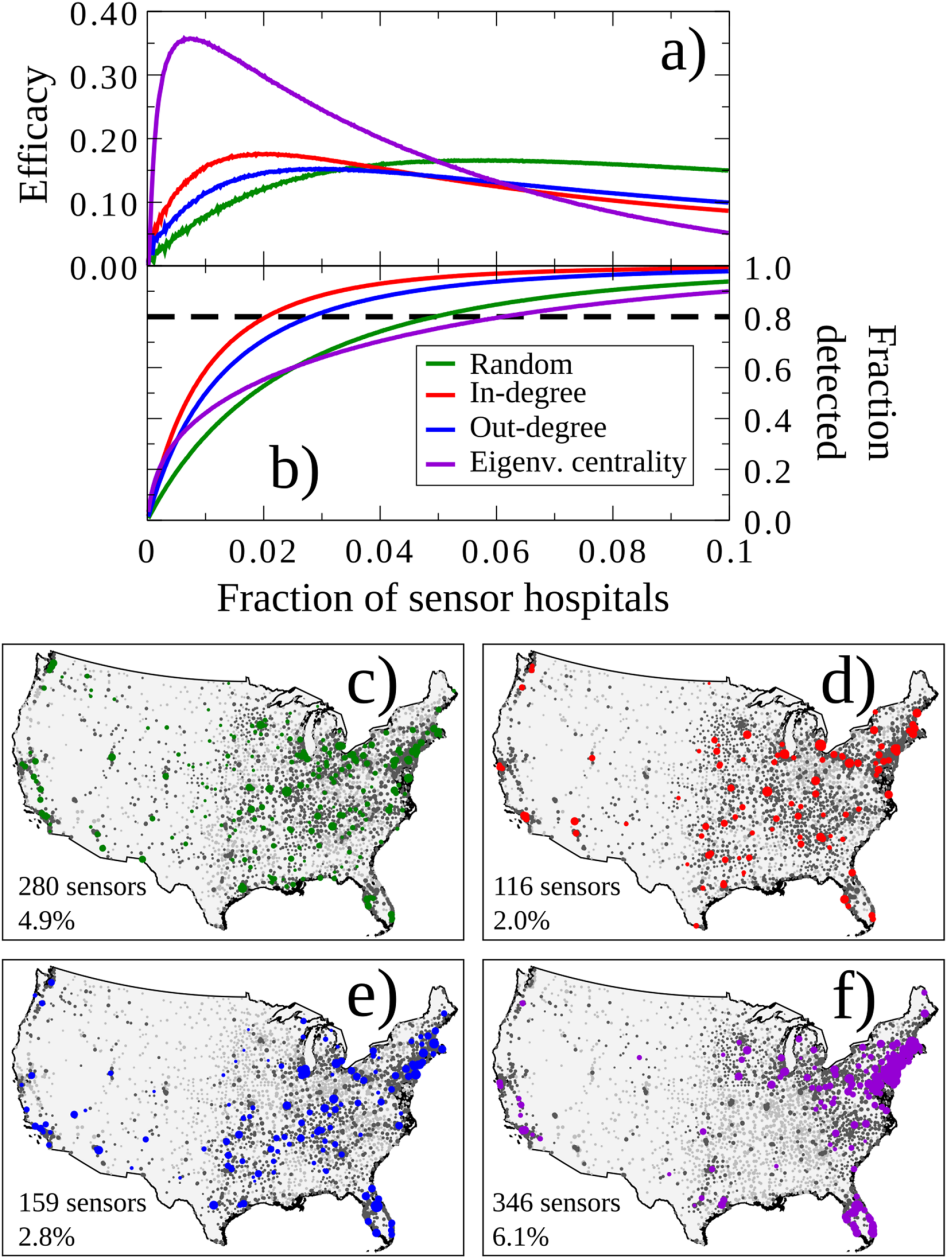
Efficacy and spatial locations of optimal sensors. (**a**) Efficacy of strategies. When focusing on the most efficacious sensor set for each strategy, eigenvector centrality results in the smallest sensor set, followed by in-degree, out-degree, and the random strategy. Sensor sets are selected using the four possible strategies at 80% coverage of *C. difficile* cases in panel (**b**). Panels (**c**, **d**, **e** and **f**) show instances of the sensor sets at 80% coverage for the different strategies (in-degree, out-degree, random, eigenvector centrality). The colored nodes are included in the sensor set, the dark gray nodes are neighbors of the sensor hospitals, and the light gray nodes are not covered by the sensor set. Hospital size is proportional to the number of *C. difficile* cases in that hospital. The in-degree strategy uses the least number of sensors and it is followed by the out-degree and then the random strategy. The eigenvector centrality strategy unexpectedly performs worse than the random strategy. The maps were created using the Basemap Matplotlib Toolkit 1.0.8 (http://matplotlib.org/basemap/) for Python^24^.

We investigated the optimal selection of network sensors for theoretical detection of emerging nosocomial epidemics, and we focus on two measures: the efficacy and fraction of detected cases for four possible selection strategies are shown in Fig. 3. The “eigenvector centrality” strategy achieved the highest efficacy using the smallest number of sensors, using only 42 hospitals, or 0.7% of all facilities. It was followed by the “in-degree” strategy, for which the maximum efficacy was achieved for 108 sensors, or 1.9%, of all facilities. The “out-degree” strategy was the third best strategy, at most using only 167 facilities, or 2.9%, as sensors. Both degree-based approaches outperformed the random strategy that uses 332 facilities, or 5.9%, as sensors. In terms of the fraction of detected cases, the degree-based and random strategies performed similarly at optimal efficacy: 78% for in-degree, 81% for out-degree, and 84% for the random strategy. In contrast, the eigenvector centrality performed very poorly at 37%. When the strategies are compared at the level with 80% of detected cases, the in-degree strategy performs the best (116 sensors, 2%), followed by the out-degree strategy (159 sensors, 2.8%), followed by the random strategy (280 sensors, 4.9%), and finally the eigenvector centrality strategy (346 sensors, 6.1%). In Fig. 3c–f, an instance for the optimal sensor set derived from each is plotted on the map. Table 2 describes the composition of the optimal sensor sets in terms of types of facilities. The eigenvector centrality strategy had significantly less proportion of general hospitals and an abundance of rehabilitation facilities (p = 0.003 by χ^2^).

**Table 2:** Configuration of optimal sensor sets.

Facility set	N (%)	% General hospitals	% Rehabilitation facilities	% Others	Coverage
All facilities	5667 (100%)	4595 (81.1%)	531 (9.4%)	541 (9.5%)	100%
**Sensor strategy**					
In-degree (max. efficacy)	108 (1.9%)	88.9%	5.6%	5.6%	78%
Out-degree (max. efficacy)	167 (2.9%)	95.2%	2.4%	2.4%	81%
Eigenv. Centrality (max. efficacy)	42 (0.7%)	78.6%	11.9%	9.5%	37%
Random (max. efficacy)	332 (5.9%)	81.1%	9.4%	9.5%	84%
In-degree (80% cov.)	115 (2.0%)	88.7%	6.1%	5.2%	80%
Out-degree (80% cov.)	158 (2.8%)	94.9%	2.5%	2.5%	80%
Eigenv. Centrality (80% cov.)	346 (6.1%)	84.1%	10.7%	5.2%	80%
Random (80% cov.)	279 (4.9%)	81.1%	9.4%	9.5%	80%

The table describes the characteristics of the optimal sensor sets (percentage of the total number of facilities, percentage of genera/rehab/other facilities) and the coverage of cases. The optimal sensors, depending on the strategy, are compared both at maximum efficacy and at an 80% coverage rate.

## Discussion

We studied a network defined by the transfer of a large number of Medicare patients across 5,667 US facilities over a 2-year period. We found the transfer network to be strongly bound by geography with 90% of all transfers spanning a distance less than 200 km. We also found that the transfer network could plausibly serve as a substrate for the spread of nosocomial infections: we observed a positive correlation among mean *C. difficile* incidences between facilities and their network neighbors. This association was significantly stronger with a hospital’s network neighbors than with nearby non-connected hospitals across a wide range of geographic distances. This result is in line with the recent work of Simmering *et al*.^14^ which demonstrated a correlation of *C. difficile* incidence with weighted and unweighted in-degree of the transfer network. Here, we consider not only topological properties of the network, but also the *C. difficile* incidence at hospitals neighboring an index hospital, and therefore more directly test the hospital-hospital infection hypothesis. In addition, we consider the role of different spatial scales and their interaction with the structure of the transfer network. Note that establishing complete certainty about the spread of a particular strain of *C. difficile* bacteria would require genetic data, which was not available in this study. Finally, we showed that selecting facilities as sensors based on their in-degree was able to detect a large fraction of infections with only 2% of the facilities acting as sensors. A key strength of our approach is its reliance on routinely collected administrative data. These results support our conceptual model of using the nationwide patient transfer network to monitor health-care associated infections, likely well beyond the illustrative case of *C. difficile* considered here. In particular, our work demonstrates the potential benefit of using a network of interconnected facilities to monitor incipient outbreaks. Of course, it is possible that different pathogens might need a different number of sensor facilities, a different set of sensor facilities, or different surveillance windows.

This study builds on prior research examining physician networks^19, 20^ and facility networks by incorporating nationwide data on hospital transfers together with epidemiologic data on healthcare associated infections by *C. difficile* ^21^ Using methods from network analysis, we find that a facility’s rate of *C. difficile* cases is significantly correlated with those of its transfer neighbors. There are two possible explanations for this phenomenon: first, that transfers can serve as a substrate for the spread of *C. difficile*, correlating the infections rates of connected hospitals, or second, that community factors driving *C. difficile* infection rates influence nearby hospitals. Prior epidemiologic research suggests that there are significant reservoirs of *C. difficile* in both hospitals and the community, therefore both of these explanations may contribute to the pattern we observe^22^. However, our findings support the hypothesis that *C. difficile* incidence is plausibly related to hospital transfers, due to the stronger association we observe between a hospital’s *C. difficile* incidence and its network neighbors than with non-connected nearby hospitals (Table 2).

Regardless of the causal mechanism of this correlation, using facilities with significant numbers of inbound transfers as “sensors” for new outbreaks of known or emerging infections could serve an important role for public health surveillance. Using different strategies to select a small group of facilities as potential “sensors,” we find that choosing facilities prioritizing those with the greatest number of inbound transfers optimizes the fraction of *C. difficile* cases detected while minimizing the number of facilities needed. These facilities serve as “hubs” for transfers from a geographically diverse set of other facilities in their region, so are optimally positioned to see the leading edge of any new infection^23, 24^. It is also notable that a disproportionate number of rehabilitation facilities form part of the optimal sensor set we describe. Rehabilitation facilities are an important reservoir for healthcare associated infections and should be considered as a crucial part of the epidemiologic network connecting the entire US inpatient care system^25^.

We wish to point out the connection between our sensor method and three different lines of related work: control theory on complex networks^26^, the vertex covering problem^27^, and Borgatti’s “key player” approach^28^. In these three problems, one tries to find a minimum set of nodes that will have a maximal global impact in the network, whether in terms of controlling some dynamical process taking place on it (control theory), finding the minimum set of nodes such that every edge of the network is incident to at least one of the nodes in the set (vertex cover), or finding the set of *k* nodes that is maximally connected to all other nodes. In our case, the proposed method attempts to find a minimum set of nodes that will cover most of the observed infected nodes. In this sense our approach is most similar to the vertex cover problem with the added nuance that we now have different numbers of observed cases within different nodes (hospitals).

Our results are in agreement with previous findings, starting from research showing that healthcare outcomes can be associated with the structure of the transfer network of patients^8–10^. The risk of *C. diff* spreading through the transfer network of patients is supported by the empirical results of Huang *et al*.^13^ and Simmering *et al*.^14^, and the by the simulation results of Lee *et al*.^12^. This literature has demonstrated that the incidence of *C. diff* in connected hospitals is associated with incidence in a central hospital, and also that the in-degree of a hospital is positively correlated with its *C. diff* incidence. Others have shown that the structure of the network is spatially constrained and heterogeneous^11^. Finally, Karkada and collaborators^7^ showed through simulation that any network-aware algorithm for resource allocation will be better than a random strategy. In their study the betweenness centrality strategy performs the best, and they also provide a greedy strategy, an approach we discuss in the SM.

Our study has several limitations. First, the data we used to map the facility networks are from 2006 and 2007. However, given that facility transfer patterns are strongly embedded in the geography of the country, we do not expect the age of the data to affect our results substantially. Second, we cannot assess the extent to which unobserved policies or commercial constraints might have affected the flow of patients from one facility to another; however, these policies merely affected patient transfers, which are observable in the current and similar future data. Third, our analyses and models assume that patient transfers are the only mechanism responsible for the spread of infections. There are, of course, other vectors or means that might result in facilities being infected, such as the movement of physicians, nurses, and other health care staff, and equipment, between facilities. In addition, our analysis is limited by our use of the Medicare population to model infection spread. Even though Medicare patients represent a substantial proportion of hospitalizations nationally, it is possible that our results may not generalize to other populations. Finally, in this analysis we did not make use of the fine-scale temporal information available in transfer data; future work could evaluate how bursts of infected patients might contribute to an epidemic.

Understanding the structure and dynamics of the facility transfer network for the spread of real infections has a number of important implications. Empirical data could be used either periodically, or in real time, to map networks of patient movement in the US health care system, and this network could then be used monitor the spread of nosocomial and other infections in the network. In our estimation, such an approach could detect 80% of *C. difficile* cases among Medicare patients using just 2% of facilities as network sensors. Furthermore, the method for choosing sensor hospitals, namely in-degree, relies only on local network measures, which provides a scalable method easy to implement dynamically without the need of global network structure knowledge. The approach is also expected to be robust even if the network data is not fully up to date, which makes the approach more useful in practice (see Robustness of sensor set performance in the SM). Finally, this approach would be useful not only for public health interventions, in the case of natural epidemics, but also in the case of deliberate ones, such as those due to a possible bioterror attack. Public health and clinical care can be enhanced through a deeper understanding of the network of health care facilities in which patients and practitioners are embedded.

## Methods

The study was approved by Harvard Medical School IRB.

### Study population

We examined inter-facility transfer patterns of the entire population of US Medicare beneficiaries over a two-year period. We used a 100% sample of the Medicare Provider Analysis and Review (MedPAR) files for calendar years 2006 and 2007. The data was anonymized and patient id’s were only used in order to match stays in different facilities in order to infer direct transfers (see subsection “Constructing the transfer network”). The MedPAR files contain demographic, diagnosis, procedure, and billing information on all inpatient and skilled nursing facility (SNF) stays divided into 144 variables. Of these variables, we only kept the patient ID, the ID of the hospital where the patient stayed, and admission and discharge dates. Our study cohort consisted of Medicare patients aged 65 or older with an initial facility stay at an acute medical or surgical facility with an active record in the American Hospital Association (AHA) 2005 database^29^. This database contains information about the facilities, summarized by 858 variables, of which we kept the Medicare provider ID (which allows us to cross-link the databases), name, address, coordinates (latitude and longitude), and the number of beds. We divided facilities into three classes: acute medical-surgical hospitals (“general hospitals”), rehabilitation facilities, and other facilities. Before applying these exclusion criteria, we identified 26.4 million stays of 12.5 million patients in 6,278 different hospitals. After the exclusions, our final cohort consisted of 10.4 million patients with 21.0 million inpatient stays in 5,667 different hospitals. We characterized comorbidities using the Charlson index^30^.

### Constructing the transfer network

We defined an inpatient transfer whenever a patient, who have unique id’s in our dataset, was discharged from one facility and admitted to another facility on the same calendar day. A minority of transfers as defined here may not correspond to actual formal transfers of patients due to the possibility of a same-day readmission; however, these are equivalent to patient transfers from an epidemiological point of view. (See Supplementary Material [SM] for a sensitivity analysis). We identified 936,101 transfer events from 741,732 patients taking place between 76,003 pairs of facilities in the calendar years 2006 and 2007.

We constructed then a network representation of the patient transfers across facilities. Facilities were represented as “nodes” and a transfer of a total of *x* patients on day *d* from facility *i* to facility *j* was represented as a directional connection, or “directed edge,” from node *i* to node *j* with weight *x* on day *d*. This longitudinal sequence of patient transfers formed a day-to-day network with “directed” edges with weights equal to the number of patients transferred on day *d* for 2 years. We constructed a static representation of the network that retained no temporal information of patient transfers by aggregating the data for the two-year period. In this aggregated network, the weight of the edge from node *i* to node *j* represents the mean daily number of patient transfers along that edge.

### Network measures

We make use of three network centrality measures: (i) in-degree, the number of incoming edges; (ii) out-degree, the number of outgoing edges; and (iii) eigenvector centrality, a measure of centrality based on the eigenvector associated with the largest eigenvalue of the network adjacency matrix. The first two measures are local in the sense that their values can be computed for any given node based on knowledge of the node’s (nearest) network neighbors. The third measure is global, and it assigns higher centrality scores to nodes that are connected to other nodes with high centrality scores, thus taking into account the overall structure of the network. Variations of this measure include the Katz centrality and the PageRank centrality.

### *Clostridium difficile* incidence on the transfer network

The transfer of contagious patients from one facility to another could result in the transmission of pathogens between them. In order to ascertain the transmission of *C. difficile* across hospitals, one would need genetic data for the pathogen, which is not available in our setting. As an alternative and more scalable approach, we examined the incidence of *C. difficile* infection and its correlation with properties of the transfer network. *C. difficile* is an anaerobic, gram-positive, spore-forming bacteria that is spread by ingestion of *C. difficile* spores, which are very hardy and can persist on environmental surfaces for months without proper hygiene^31^. We ascertained incident cases of *C. difficile* infection by identifying any facility admissions with ICD-9 diagnostic code 008.45 in any field (upon discharge). The sensitivity and specificity of using ICD-9 codes to identify *C. difficile* infections have been reported by multiple groups to be adequate for identifying overall *C. difficile* burden for epidemiological purposes^29, 32, 33^.

We calculated the *C. difficile* incidence at each facility, defined as the number of patients that were admitted to the facility with that particular discharge diagnosis over the study period divided by the total number of patients admitted to the facility over the same period, and plotted the *C. difficile* incidence of the facility against the average incidence of *C. difficile* in facilities connected to that facility in the transfer network. We stratified the plot by facility type (general hospital, rehabilitation facility, other, see SM), and quantified the correlation using the Pearson linear correlation coefficient.

Any correlation in *C. difficile* incidence that we observe could be potentially driven by a common reservoir in the geographical vicinity of the hospitals rather than the spread of infections via hospital transfers. Ideally, we could assess genetic markers to assess whether the same strains of *C. difficile* flowed from one hospital to another via patient transfer. Because these data are not available, we instead assessed the contribution of patient transfers by hypothesizing that if transfers are a causal mechanism for *C. difficile* spread, then there should be a stronger association between *C. difficile* incidence in nearby hospitals connected by patient transfers than in nearby non-connected hospitals.

More formally, we used fractional logistic regression, or logistic regression using proportions as outcomes^34^, to predict the outcome of the incidence of *C*. *difficile* at a hospital *i*, *ρ*_*i*_, using a model with two separate predictors: (1) the average incidence at connected (through the transfer network) hospitals within less than a certain distance *D*, ${\langle \rho \rangle }_{i}^{Net}(D)$, compared to (2) the average incidence at all hospitals less than that distance *D*, ${\langle \rho \rangle }_{i}^{com}(D)$. With this the model is specified as

$$log\frac{{\rho }_{i}}{1-{\rho }_{i}}=\alpha +\beta {\langle \rho \rangle }_{i}^{Net}(D)+{\rm{\gamma }}{\langle \rho \rangle }_{i}^{Com}(D)$$

(see SM for more details). If hospital transfers are an important mechanism for *C. difficile* transmission, then the regression coefficient for ${\langle \rho \rangle }_{i}^{Net}(D)$, β, should be greater than the coefficient for ${\langle \rho \rangle }_{i}^{Net}(D)$, γ, across all distances as a predictor of a given hospital’s *C. difficile* incidence. We standardized the quantities ${\langle \rho \rangle }_{i}^{Net}(D)$ and ${\langle \rho \rangle }_{i}^{com}(D)$ as z-scores (e.g. we subtracted the mean and divided by the standard deviation for each quantity) in order to account for different ranges in the different indicators (see SM for further inspections).

### Sensor placement on the facility network

Given that patient transfers form an interconnected system of facilities, it might be possible to make use of the properties of the transfer network to set up a surveillance system for infections, such as theoretically new antibiotic-resistant organism. For this application, although exhaustive data could be available for all facilities all the time (real-time reporting), it may not be feasible for infections which show non-specific symptoms and for which testing methods require considerable cost. Therefore, this limitation calls for a parsimonious approach in which only a subset of facilities is monitored at any given time. We call these monitored facilities “network sensors” in the sense that they could be used to sense incipient epidemics in the entire network. We consider three different active strategies for sensor selection and one control strategy: (1) choose sensor facilities in proportion to their in-degree; (2) choose sensor facilities in proportion to their out-degree; (3) choose sensor facilities in proportion to their (undirected) eigenvector centrality; and (4) choose sensor facilities uniformly at random from the set of all facilities (the control strategy). The first two strategies rely on the most basic local centrality network measures and require only minimal information about network structure. The third one relies on a global network measure, and as such it might be more difficult to implement in practice. The control strategy serves as a baseline in the absence of any network data. We take a stochastic approach in the selection of the sensor nodes, i.e., sensors are chosen proportionally to their in-/out-degree or eigenvector centrality, in order to overcome the fluctuations present in network measures due to the temporal nature of the data. In the following simulations, we assume that a sensor facility is able to detect every infected patient who is in the facility itself or in any of the facilities to which the facility is connected via patient transfers. While this assumption is made primarily for methodological convenience and may not hold in practice, the relative performance of the three strategies for selecting sensors remains unaffected if this assumption is relaxed. The choice of in-degree and out-degree is made because these measures represent the most basic metrics for determining the centrality of a node in a directed network.

### Determining the optimal sensor set

We define the relative efficacy of the sensor *E*_*N*_ set as

1 $${E}_{N}=\frac{{D}_{N}}{N{D}_{1}}-\frac{M-{D}_{N}}{M}$$

where *N* is the number of sensors in the sensor set (based on the strategies outlined above), *D*_*1*_ and *D*_*N*_ are the average numbers of infected patients detected by sensor sets of size 1 and *N*, respectively, and *M* is the total number of *C*. *difficile* cases in all of the facilities combined. The choice of sensors in all the strategies is made randomly in proportion to the measure that defines the given strategy (except for the random strategy, where the choice is made purely at random), and the number of detected cases is defined as an average taken over different realizations of sensor sets of the same size. For *D*_*1*_, only one sensor is chosen in proportion to the chosen metric, such as eigenvector centrality, and the average is computed over 10000 such selections. While each additional sensor always improves the overall performance of the system, denoted by *D*, any sensor set exhibits diminishing marginal returns. The first term in the definition of the efficacy corresponds to the number of detected cases normalized by the number of cases that would be detected if all sensors in a sensor set of size N were as efficacious as the sensor in the set consisting only of one sensor (*ND*_*1*_). The second term is a penalty term that corresponds to the fraction of undetected cases. High relative efficacy is therefore a combination of selecting a set of sensors that are as close as possible in efficaciousness to the first sensor and having these sensors miss as few cases as possible.

Since we know the number of *C. difficile* cases in each facility at any given time, we can simply count the number of cases in the sensor facilities and their network neighbors. We average the results by generating 10,000 independent realizations of sensor sets for each of the four different strategies of choosing sensors (in-degree, out-degree, eigenvector centrality, random). The optimal sensor set for each strategy is the one with maximum efficacy. This measure informs us about the best way of allocating sensors when resources are severely limited. In addition, we consider sensor sets that cover as many cases as possible, which is the reason for comparing sensor sets at the 80% level of detected cases (the second term).

### Statistical analysis

Univariate comparisons were assessed using χ^2^ tests. Bivariate associations were assessed with Pearson correlation coefficients and bivariate regression. For the statistical tests and simulations in these analyses, we used SAS, version 9.4 (Cary, NC), Fortran and Python, including the scipy.stats and statsmodels package in Python.

### Plotting maps

The maps were created using the Basemap Matplotlib Toolkit 1.0.8 (http://matplotlib.org/basemap/) for Python^35^.

### Data Availability Statement

The data used in this study are available from the Centers for Medicare and Medicaid Services (CMS), but fees and restrictions may apply to their use.

## Electronic supplementary material

Supplementary information
