# Supplementary material for: Influence of a patient transfer network of US inpatient facilities on the incidence of nosocomial infections
Source: Sci Rep. 2017 Jun 7;7:2930. doi: 10.1038/s41598-017-02245-7 (PMC5462812; doi:10.1038/s41598-017-02245-7)
Supplement: Supplementary file 1 — Supplementary information [file 41598_2017_2245_MOESM1_ESM.pdf]

Supplementary Material for: Influence of a patient transfer network of US inpatient facilities on the incidence of nosocomial infections

Juan Fernández-Gracia*, Jukka-Pekka Onnela*, Michael Barnett, Víctor M. Eguíluz, and Nicholas A. Christakis

Affiliations: Institute for Cross-Disciplinary Physics and Complex Systems (JFG and VME); Harvard T. H. Chan School of Public Health (JFG and JPO) Brigham and Women's Hospital (MB); and Department of Medicine, Department of Sociology, and Yale Institute for Network Science, Yale University (NAC).

TABLE OF CONTENTS

SUPPLEMENTARY METHODS AND RESULTS 1
Transfer network 1
Robustness of the transfer extraction 2
***C. difficile* incidence correlation by hospital type** 2
Finding a threshold for the two regimes in *C. difficile* incidence correlation for general hospitals 2
Adjusting for hospital size 3
Community acquired infections vs hospital acquired infections 3
Optimal sensor set 3
Robustness of sensor set performance 4
List of hospitals included in the sensor sets 4
In-degree strategy 4
Out-degree strategy 5
Greedy algorithm 5
TABLES 7
FIGURES 8
REFERENCES 16

SUPPLEMENTARY METHODS AND RESULTS

Transfer network

We examine the structural connectivity and geographic characteristics of the static transfer network in Fig. S1. In terms of network topology, the in-degree distribution has a broader tail than the out-degree distribution. The network has an average (local) clustering coefficient of 0.51. This coefficient measures the probability that any two hospitals connected to an index hospital are in turn connected to each other, forming a closed triad (a cycle of three nodes and three edges). A random graph with the same number of nodes and edges yields an average local clustering coefficient of 0.0057 ± 0.0001 (SE), which is substantially lower than the observed value, a finding that likely reflects the network's geographic embeddedness. The average shortest path length of the network is 4.69. To put this number in perspective, we performed network randomizations using a slight variant of the directed configuration model that preserves both in-degree and out-degree distributions[S1]. This approach gave rise to an average shortest path length of 3.6 ± 0.4 (SE). The observed network is therefore a somewhat "larger world" than what would be expected by chance, but this is almost certainly driven by the underlying geography and the objective of keeping transfers as short as possible. In fact, about 90% of the transfers are to hospitals less than 200km away.

Degree assortativity is the concept that nodes with many connections tend to be connected to other nodes with many connections[S2,S3]. When the static network is taken as undirected, we can use the assortativity coefficient to measure the extent to which the degrees of hospitals in each pair of connected hospitals are similar. We obtain a slightly negative value of -0.06, but similar values of -0.005 ± 0.001 (SE) also arise from randomizations of the network using the algorithm discussed above. Consequently, there is no statistically significant assortativity in the network over and above what would be expected by chance given the network's degree distributions.

Robustness of the transfer extraction

Since the patient transfers are not explicit in the data but instead need to be inferred from the data, we investigated the robustness of some of the results to our definition of what constitutes a hospital transfer. Instead of requiring readmission on the day of discharge, we relaxed this definition by allowing the readmission to take place also on the day after discharge. A visual examination of Fig. S2 shows that the edges induced by the same-day rule (red edges) and the additional edges that result using the relaxed rule (blue edges). This relaxation leads to 67472 additional transfers (7.2% increase). There are 11827 new edges that appear on the transfer network (15.6% increase), with an average transfer load of 1.2 with a standard deviation of 0.7. For the connections that appear under both rules, the difference in transfer loads averages to 0.7 transfers with a standard deviation of 1.9. The distribution of edge weights for both cases are shown in the upper left panel of Fig. S3, and the two distributions appear visually very similar to one another. The weight distribution of the additional edges, as well as the distribution of weight differences for the common edges in both cases can be seen in the upper right panel of Fig. S3. The range of this distribution is much more constrained than that of the actual weight distributions. The number of transfers increases, but the deviations are minimal. Note also that both measures of transfers are strictly speaking wrong, as the first one based on the one-day rule is really a lower bound on the number of transfers and the second one (based on the relaxed rule) is an upper bound. Given the similarity of these findings across the two rules, in the following we work with the lower bound (same day discharge and readmission).

C. difficile incidence correlation by hospital type

In order to gain more insight about the influence of different types of facilities we divided the hospitals into three categories, namely general hospitals, rehabilitation facilities and other hospitals. We plot in Fig. S4 the *C. difficile* incidence vs. the average *C. difficile* incidence in the neighborhood.
Two separate "regimes" of correlation were apparent for general hospitals (Fig. S4a, 4546 out of 5667 hospitals), separated by a mean hospital incidence of 3.1%. The high incidence regime is populated by just 149 hospitals. In the low incidence regime, the two variables are reasonably strongly correlated (Pearson correlation coefficient 0.50, 95% CI: 0.48, 0.52), whereas in the high incidence regime there is no correlation (correlation coefficient -0.07, 95% CI: -0.23, 0.09). Linear regression of the average mean incidence in neighboring hospitals on incidence in the case hospital reveals a similar trend, where the regression coefficient is estimated as 0.53±0.02 in the low incidence regime and -0.03±0.04 in the high incidence regime. The regression coefficient measures the average magnitude of the correlation. So if the regression coefficient is 0.53, it means that a variation of 1% in *C. difficile* incidence will translate on average in a 0.53% variation in the neighboring hospitals. In contrast to general hospitals, the plot reveals just one regime for rehabilitation hospitals (Fig. S4b, 526 hospitals), where the correlation coefficient is 0.33 (95% CI: 0.25, 0.40) and the estimated slope in the linear regression is 0.049±0.006. The correlation in incidences for the remaining hospitals (Fig S4c, 535 hospitals) was weaker (0.18, 95% CI: 0.09, 0.26) with the slope estimated as 0.10±0.02.

Finding a threshold for the two regimes in *C. difficile* incidence correlation for general hospitals

Although in the main text we showed only the data for C. difficile incidence below 0.05 for general hospitals, when plotting the *C. difficile* incidence versus the average mean *C. difficile* incidence in the hospital neighborhood we found two regimes for the full range. In order to objectively give a cut-off separating the two regimes we computed the Pearson correlation coefficient of the two regimes as a function of the cut-off and kept the value for which the correlation coefficient of the low incidence cloud was maximum. See Fig. S5.

Adjusting for hospital size

It could be argued that hospital size, as a proxy for its activity, would be a major indicator of *C. difficile* incidence. In order to control for hospital size, we adjusted three different models to the data of *C. difficile* incidence in the low incidence regime for general hospitals, which is the one showing the strongest signal (see the two previous sections). The first model is a logistic regression of *C. difficile* incidence using as indicator the average *C. difficile* incidence in the network neighborhood of the central hospital, the second model uses hospital size as indicator and the third model uses both. The complete model is written as

$$\log \frac{\rho_i}{1-\rho_i} = \alpha + \beta \langle \rho \rangle_i + \gamma\, s_i/100 \quad ,$$

where ρ_i is the incidence at hospital *i*, $\langle \rho \rangle_i$ is the average incidence at the network neighborhood of hospital *i* and $s_i/100$ is the size of hospital *i* in number of beds divided by 100. The main result is that the effect of size per 100 beds is an increase in 7% in the odds of *C. difficile* incidence, while for a 10% increase in the average C. difficile in the neighborhood the effect is a 3000% increase in the odds of *C. difficile* incidence. Thus network neighborhood has a much stronger effect on *C. difficile* incidence. Results are shown in Table S2.

Community acquired infections vs hospital acquired infections

In order to know if the correlation in *C. difficile* incidence is due to transfers of patients between hospitals or because the infections come from a common reservoir in the geographical vicinity of the hospitals, one should be able to access genetic data in order to assess that the same strain is flowing from a hospital to another one. These data are not available in our case. At most we can show that the incidence at a hospital *i*, ρ_i is more correlated with the hospitals that are at less than a certain distance *D* and connected through the transfer network, $\langle \rho \rangle_i^{Net}(D)$, than with the average incidence of all the hospitals that are at less than that distance *d*, $\langle \rho \rangle_i^{Com}(D)$. The logistic regression looks like

$$\log \frac{\rho_i}{1-\rho_i} = \alpha + \beta \langle \rho \rangle_i^{Net}(D) + \chi \langle \rho \rangle_i^{Com}(D) \quad .$$

We checked that the indicators are not extremely correlated by computing their Pearson correlation coefficient as a function of distance *D*. as can be seen in Fig.S6A
The results of the coefficients as a function of the distance *d* are shown in Fig. S6B. The inset shows the value R^2 associated with the regression also as a function of the distance *D*. The results show that the effect associated to hospitals which are connected through the transfer network saturates around 40 from 100km on, while the effect of the average of all hospitals up to a certain distance is a growing function of *D* which is less important for distances below 250km, but reaches the effect of connected hospital from there on. In conclusion the average incidence in network neighbor hospitals is substantially more representative of a hospitals own incidence than the incidence in the geographical vicinity unconstrained with the transfer network for distances smaller than 250km and both effects are comparable at larger distances.

We also standardized the values of $\langle \rho \rangle_i^{Net}(D)$ and $\langle \rho \rangle_i^{Com}(D)$, by subtracting their means and dividing by their standard deviation. In this way we have comparable ranges among all variables. The different values of the standard deviation for the network and community indicators are shown in Fig.S6c. Then we applied the same regression technique to obtain the results in FigS6d, which corroborate our finding in Fig.S6b that the network neighbors have a stronger influence on the incidence of a hospital than those hospitals at similar distances but not connected via transfers and extends the results to all the range of distances explored.

Optimal sensor set

We determine the best sensor set we could have possibly chosen given the observed data. In order to do this, we use greedy algorithms,[S4] as checking all possible combinations of hospitals to use as sensors grows exponentially in the number of hospitals and is therefore not feasible for any but the smallest hospital transfer networks. For a fast algorithm that is not guaranteed to give the optimal answer (as is true with any heuristic algorithm), we choose the sensors sequentially. We first compute the number of

cases each hospital would detect and we choose the one that will detect the highest number of cases. We then re-compute how many new cases would be covered by each subsequent hospital if added to the existing sensor set. This continues until we find the sensor set that covers all cases. As mentioned above, this procedure does not guarantee that we will choose the optimal sensor set given a number of sensors *N*, but it is however very efficient and yields an effective sensor set not far from the optimal one. In order to check that our solution is sufficiently close to the actual best solution, we used simulated annealing[S5]. The simulated annealing procedure is suitable for optimization problems of large scale, especially ones where a desired global extremum is hidden among many, poorer, local extrema. There is an objective function to be minimized, in our case the coverage of cases to be maximized, but the space over which that function is defined is not simply the *N*-dimensional space of *N* continuously variable parameters. Rather, it is a discrete, but very large, configuration space with the number of elements factorially large, so that they cannot be explored exhaustively. This method ensures that the global maximum (or minimum depending on what is searched) is obtained, although it may take a diverging time. We checked that we found the same result for slower and slower implementations of the method, so that we can trust the result to be the global extremum. The result of this method is in agreement with the result of the fast sequential algorithm.

In Fig. S7 we show the results of finding the sensor set that maximizes the number of detected cases in the training dataset for the static network case. This method is data-based and tries to maximize the number of detected cases without the use of any strategy of choosing sensors other than the optimization procedure. In this case we find that for a very small number of 26 (0.46%) sensors, we can detect 88% of the cases. This very high performance is however likely a consequence of over-fitting the model to the observed (training) data. Using this set of hospitals as sensors for a new dataset on patient transfers would likely result in lower (and more variable) performance of the sensor system.

Finally, in Fig. S8 we can see a map depicting the sensor set that is the result of the optimization for the aggregated case.

Robustness of sensor set performance

The performance of statistical methods is generally quantified using some error metric, and most fitting procedures attempt to minimize this error in the process of finding suitable values for model parameters. It is often possible to reduce this training error by increasing model complexity, but generally the goal of modeling is to have the model perform well on a test data set, ideally an independent data set, that the model was not trained on. Good performance on a test data set, quantified by a low test error, generally leads to better overall model performance and avoids the problem of over fitting, which refers to the model adapting to the test data “too well” at the expense of poor generalizability to different realizations of data from the same data generating mechanism.

In analogy with this approach to statistical learning, we performed a series of analyses to investigate the performance of sensor sets derived from one set of data and tested on another. The objective of the analysis is twofold. First, it will enable us to ascertain the validity of our methods when applied to training data, i.e., data not used to select the set of sensors. Two, given that there are likely temporal correlations in the data, it enables us to study the performance of sensor sets on data that are temporally far removed from the training data.

Here we divided our data to disjoint (non-overlapping) windows of width *L*, where we used values of 1 month, 2 months, 4 months, 6 months, and a year for *L*. For any given window, we take the first window to be our training data and use all subsequent windows as different realizations of test data. We used the training data for generating the sensor sets (based on in-degree, out-degree, and the greedy algorithm; we exclude considerations of the random strategy here because there is no real distinction between testing and training) and evaluated the relative efficacy and the percentage of cases detected separately for each test data window.

Although intuitively it seems that the sensor sets would perform worse the greater the temporal separation between the training window and test window, we found that our methods were robust against this separation. Little variation is observed as the validation window gets more and more separated temporally from the training window that was used to construct the sensor sets (see Figs. S9-S11). This is counterintuitive especially for the sensor set obtained using the greedy algorithm because in principle we

are over-fitting our model to the data and consequently this should result in more variability. Nevertheless, temporal correlations in the dynamics of the system make it well behaved in this sense. An important lesson here is that it is possible to determine efficient sensor sets even using outdated data.

List of hospitals included in the sensor sets

In this section we list the first 26 hospitals included in the in-degree and out-degree strategies, as well as those that arise from the greedy optimization approach.

In-degree strategy

1. Saint Mary’s Hospital, 1216 Second Street SW, Rochester, MN, kin=346, kout=103
2. Cleveland Clinic Foundation, 9500 Euclid Avenue, Cleveland, OH, kin=286, kout=145
3. New York-Presbyterian Hospital, 525 East 68th Street, Manhattan, NY, kin=214, kout=118
4. Mount Sinai Hospital, One Gustave L Levy Place, Manhattan, NY, kin=169, kout=80
5. St Luke's Episcopal Hospital, 6720 Bertner Avenue, Houston, TX, kin=163, kout=91
6. Barnes-Jewish Hospital, 1 Barnes-Jewish Hosp Plaza, St. Louis, MO, kin=162, kout=78
7. Massachusetts General Hospital, 55 Fruit Street, Boston, MA, kin=159, kout=88
8. Emory University Hospital, 1364 Clifton Road NE, Atlanta, GA, kin=151, kout=71
9. Methodist Hospital, 6565 Fannin Street, Houston, TX, kin=151, kout=72
10. University of Alabama Hospital, 619 South 19th Street, Birmingham, AL, kin=147, kout=79
11. Johns Hopkins Hospital, 600 North Wolfe Street, Baltimore, MD, kin=146, kout=74
12. UPMC Presbyterian, 200 Lothrop Street, Pittsburgh, PA, kin=146, kout=89
13. Brigham and Women's Hospital, 75 Francis Street, Boston, MA, kin=142, kout=91
14. Northwestern Memorial Hospital, 251 East Huron Street, Chicago, IL, kin=141, kout=75
15. Hospital of the Univ of PA, 3400 Spruce Street, Philadelphia, PA, kin=139, kout=81
16. Clarian Health Partners, I-65 at 21st Street, Indianapolis, IN, kin=136, kout=81
17. New York Univ Medical Center, 550 First Avenue, Manhattan, NY, kin=135, kout=46
18. Kessler Institute for Rehab, 1199 Pleasant Valley Way, Newark, NJ, kin=133, kout=51
19. Mem Sloan-Kettering Cancer Ctr, 1275 York Avenue, Manhattan, NY, kin=133, kout=52
20. Duke University Hospital, Erwin Road, Durham, NC, kin=132, kout=67
21. Rochester Methodist Hospital, 201 West Center Street, Rochester, MN, kin=131, kout=27
22. Vanderbilt Univ Medical Center, 1211 22nd Avenue South, Nashville, TN, kin=131, kout=77
23. Baylor Univ Medical Center, 3500 Gaston Avenue, Dallas, TX, kin=131, kout=72
24. Abbott Northwestern Hospital, 800 East 28th Street, Minneapolis, MN, kin=126, kout=24
25. Thomas Jefferson Univ Hospital, 111 South 11th Street, Philadelphia, PA, kin=124, kout=75
26. Lenox Hill Hospital, 100 East 77th Street, Manhattan, NY, kin=123, kout=63

Out-degree strategy

1. Cleveland Clinic Foundation, 9500 Euclid Avenue, Cleveland, OH, kout=145, kin=286
2. New York-Presbyterian Hospital, 525 East 68th Street, Manhattan, NY, kout=118, kin=214
3. Saint Marys Hospital, 1216 Second Street SW, Rochester, MN, kout=103, kin=346
4. Brigham and Women's Hospital, 75 Francis Street, Boston, MA, kout=91, kin=142
5. St Luke's Episcopal Hospital, 6720 Bertner Avenue, Houston, TX, kout=91, kin=163
6. UPMC Presbyterian, 200 Lothrop Street, Pittsburgh, PA, kout=89, kin=146
7. Univ of TX M D Anderson Ctr, 1515 Holcombe Boulevard, Houston, TX, kout=89, kin=114
8. Massachusetts General Hospital, 55 Fruit Street, Boston, MA, kout=88, kin=159
9. UCSF Medical Center, 500 Parnassus Avenue, San Francisco, CA, kout=81, kin=107
10. Clarian Health Partners, I-65 at 21st Street, Indianapolis, IN, kout=81, kin=136
11. Hospital of the Univ of PA, 3400 Spruce Street, Philadelphia, PA, kout=81, kin=139
12. Mount Sinai Hospital, One Gustave L Levy Place, Manhattan, NY, kout=80, kin=169
13. University of Alabama Hospital, 619 South 19th Street, Birmingham, AL, kout=79, kin=147
14. Atlanticare Regional Med Ctr, 1925 Pacific Avenue, Camden, NJ, kout=79, kin=13
15. Barnes-Jewish Hospital, 1 Barnes-Jewish Hosp Plaza, St. Louis, MO, kout=78, kin=162
16. Vanderbilt Univ Medical Center, 1211 22nd Avenue South, Nashville, TN, kout=77, kin=131
17. Florida Hospital, 601 East Rollins Street, Orlando, FL, kout=76, kin=57
18. Shands at the Univ of Florida, 1600 SW Archer Road, Gainesville, FL, kout=75, kin=106
19. Northwestern Memorial Hospital, 251 East Huron Street, Chicago, IL, kout=75, kin=141
20. Thomas Jefferson Univ Hospital, 111 South 11th Street, Philadelphia, PA, kout=75, kin=124

21. Johns Hopkins Hospital, 600 North Wolfe Street, Baltimore, MD, kout=74, kin=146
22. Baylor Univ Medical Center, 3500 Gaston Avenue, Dallas, TX, kout=72, kin=131
23. Methodist Hospital, 6565 Fannin Street, Houston, TX, kout=72, kin=151
24. Naples Community Hospital, 350 Seventh Street North, Fort Myers, FL, kout=71, kin=27
25. Emory University Hospital, 1364 Clifton Road NE, Atlanta, GA, kout=71, kin=151
26. Memorial Hermann Hospital, 6411 Fannin, Houston, TX, kout=71, kin=114

Greedy algorithm

1. Cleveland Clinic Foundation, 9500 Euclid Avenue, Cleveland, OH, kin=286, kout=145
2. New York-Presbyterian Hospital, 525 East 68th Street, Manhattan, NY, kin=214, kout=118
3. Saint Marys Hospital, 1216 Second Street SW, Rochester, MN, kin=346, kout=103
4. Johns Hopkins Hospital, 600 North Wolfe Street, Baltimore, MD, kin=146, kout=74
5. Massachusetts General Hospital, 55 Fruit Street, Boston, MA, kin=159, kout=88
6. Univ of TX M D Anderson Ctr, 1515 Holcombe Boulevard, Houston, TX, kin=114, kout=89
7. Barnes-Jewish Hospital, 1 Barnes-Jewish Hosp Plaza, St. Louis, MO, kin=162, kout=78
8. Shands at the Univ of Florida, 1600 SW Archer Road, Gainesville, FL, kin=106, kout=75
9. UCLA Medical Center, 10833 Le Conte Avenue, Los Angeles, CA, kin=116, kout=54
10. Northwestern Memorial Hospital, 251 East Huron Street, Chicago, IL, kin=141, kout=75
11. Hospital of the Univ of PA, 3400 Spruce Street, Philadelphia, PA, kin=139, kout=81
12. Duke University Hospital, Erwin Road, Durham, NC, kin=132, kout=67
13. Baylor Univ Medical Center, 3500 Gaston Avenue, Dallas, TX, kin=131, kout=72
14. Emory University Hospital, 1364 Clifton Road NE, Atlanta, GA, kin=151, kout=71
15. UCSF Medical Center, 500 Parnassus Avenue, San Francisco, CA, kin=107, kout=81
16. St Joseph's Hosp \& Med Center, 350 West Thomas Road, Phoenix, AZ, kin=58, kout=43
17. Clarian Health Partners, I-65 at 21st Street, Indianapolis, IN, kin=136, kout=81
18. Univ of Michigan Hospitals, 1500 East Medical Center Drive, Ann Arbor, MI, kin=113, kout=53
19. UPMC Presbyterian, 200 Lothrop Street, Pittsburgh, PA, kin=146, kout=89
20. Vanderbilt Univ Medical Center, 1211 22nd Avenue South, Nashville, TN, kin=131, kout=77
21. Univ of Washington Medical Ctr, 1959 NE Pacific St, Box 356151, Seattle, WA, kin=74, kout=31
22. University of Kansas Hospital, 3901 Rainbow Boulevard, Kansas City, MO, kin=95, kout=44
23. Jackson Memorial Hospital, 1611 NW 12th Avenue, Miami, FL, kin=65, kout=51
24. OU Medical Center, 1200 Everett Drive, Oklahoma City, OK, kin=69, kout=43
25. University of Alabama Hospital, 619 South 19th Street, Birmingham, AL, kin=147, kout=79
26. University of Virginia Med Ctr, Jefferson Park Avenue, Charlottesville, VA, kin=78, kout=48

TABLES

Table s1 – Characteristics of transferred patients

	**Transferred Patients**
Patients (n)	741,732
Age (Mean (SD))	77·3 (7·6)
Female (%)	55·8
Race (%)	
White	85·5
Black	9·8
Other	4·7
Charlson score (Median, IQR)	2 (1-4)
Charlson Chronic Illnesses (%)	
Congestive heart failure	30·5
Chronic pulmonary disease	26·7
Diabetes mellitus, mild or moderate	21·7
Cerebrovascular disease	19·9
Myocardial infarction	19·0
Renal disease	16·9
Peripheral vascular disease	11·6
Any malignancy	10·5
Diabetes mellitus, severe	8·5
Dementia	5·9
Hemiplegia or paraplegia	4·1
Metastatic solid tumor	4·1
Peptic ulcer disease	2·9
Rheumatologic disease	2·8
Mild liver disease	1·3
Moderate or severe liver disease	0·8
AIDS	0·04

Abbreviations: Acquired immunodeficiency syndrome (AIDS), interquartile range (IQR), standard deviation (SD).

Table S2: Adjusting for hospital size. Parameters of the regressions of C. difficile incidence with average *C. difficile* incidence in the network neighborhood and hospital size as indicators. $\rho_i = \alpha + \beta \langle \rho \rangle_i + \gamma s_i / 100$. All fitted parameters have a $P<10^{-5}$.

Parameter	Model 1	Model 2	Model 3
α	-5.30 (±0.05)	-4.80 (±0.03)	-5.38 (±0.04)
β	37 (±3)	-	34 (±2)
ɣ	-	0.100 (±0.012)	0.07 (±0.01)
R^2	0.2	0.06	0.22

FIGURES

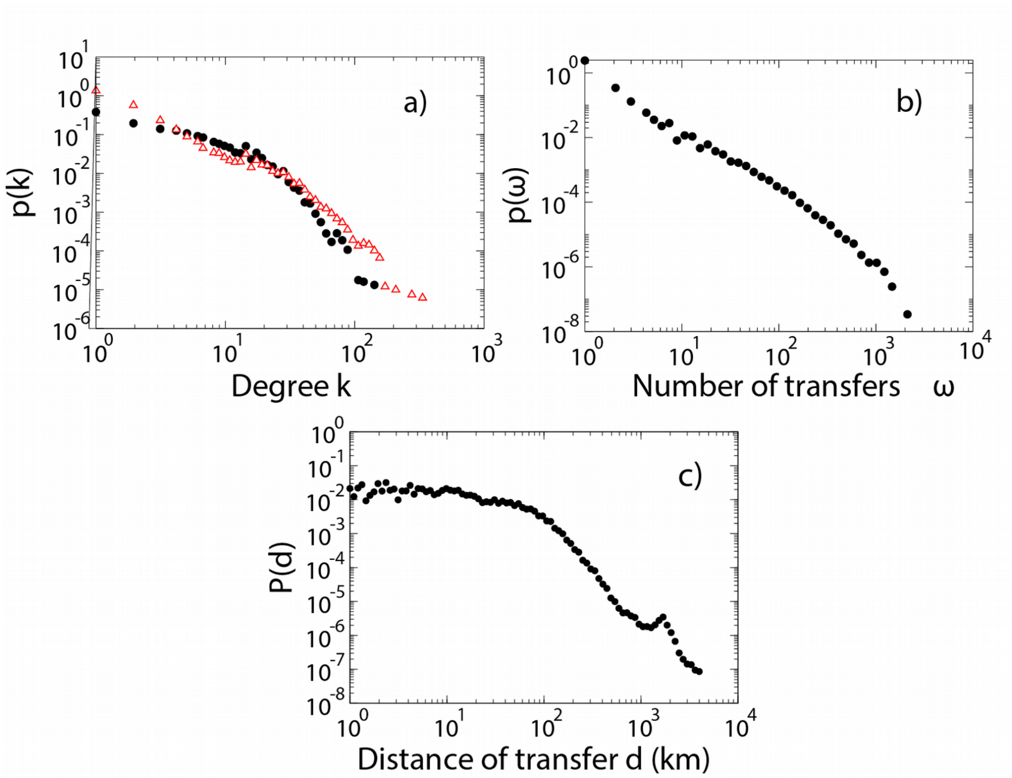

Fig. S1 Topological and geographical characteristics of the transfer network. a) Distributions for in- (red open triangles) and out-degree (black solid circles). **b)** Distribution for the number of transfers per connection ω. **c)** Transfer distance distribution.

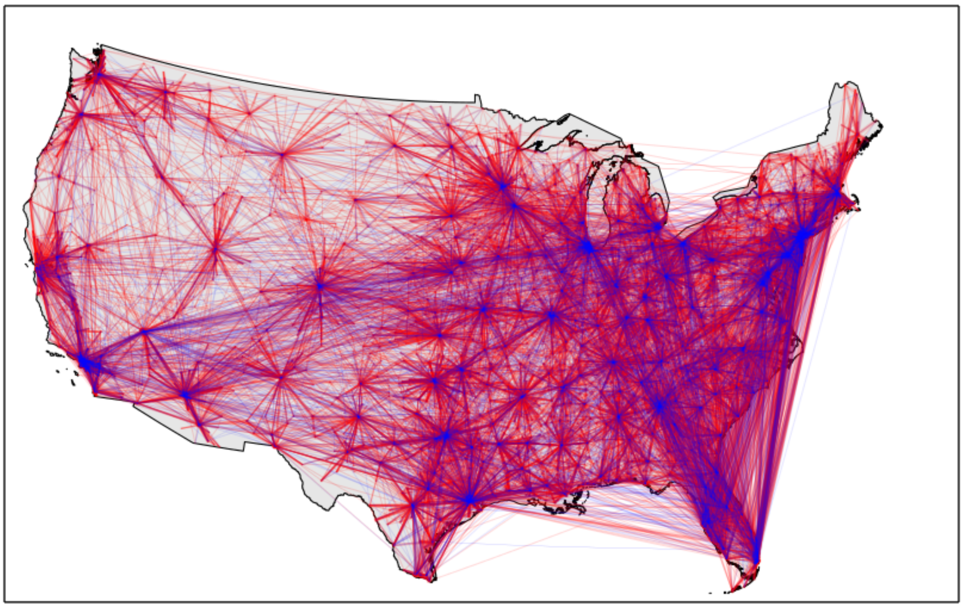

Fig S2. Comparison of the transfer network based on the 1-day and 2-day rules. The network is constructed by aggregating transfer data over the full two-year period. Red edges correspond to the connections induced by the 1-day rule and the blue edges correspond to the additional edges that appear when considering the 2-day rule. The maps were created using the Basemap Matplotlib Toolkit 1.0.8 (http://matplotlib.org/basemap/) for Python [S6].

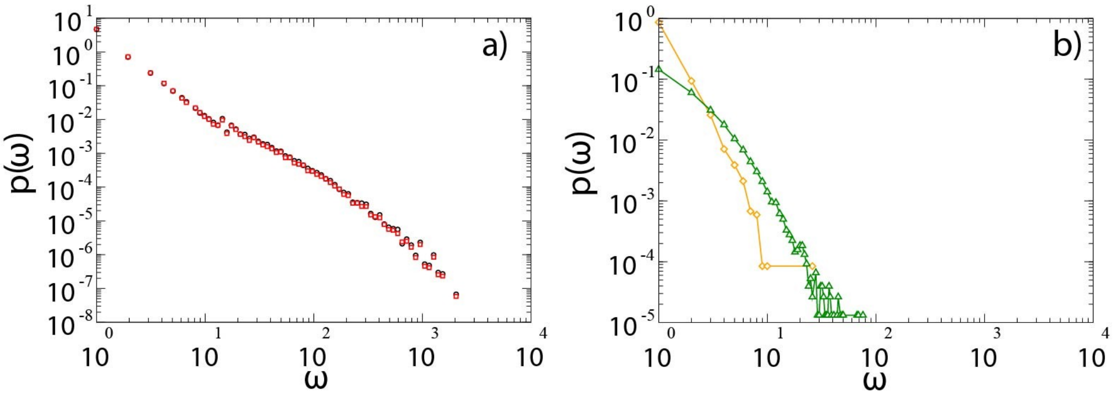

Fig S3. Comparison of transfer window of one and two days. a) Distributions of the number of transfers per connection ω in black for one day transfers (1-day rule) and in red for one or two day transfers (2-day rule). **b)** Distribution of the number of transfers per connection for the edges that appear when using the 2-day rule. Two-day transfers (orange diamonds) and of the difference in the number of transfers for the connections that are shared by the two rules (green triangles).

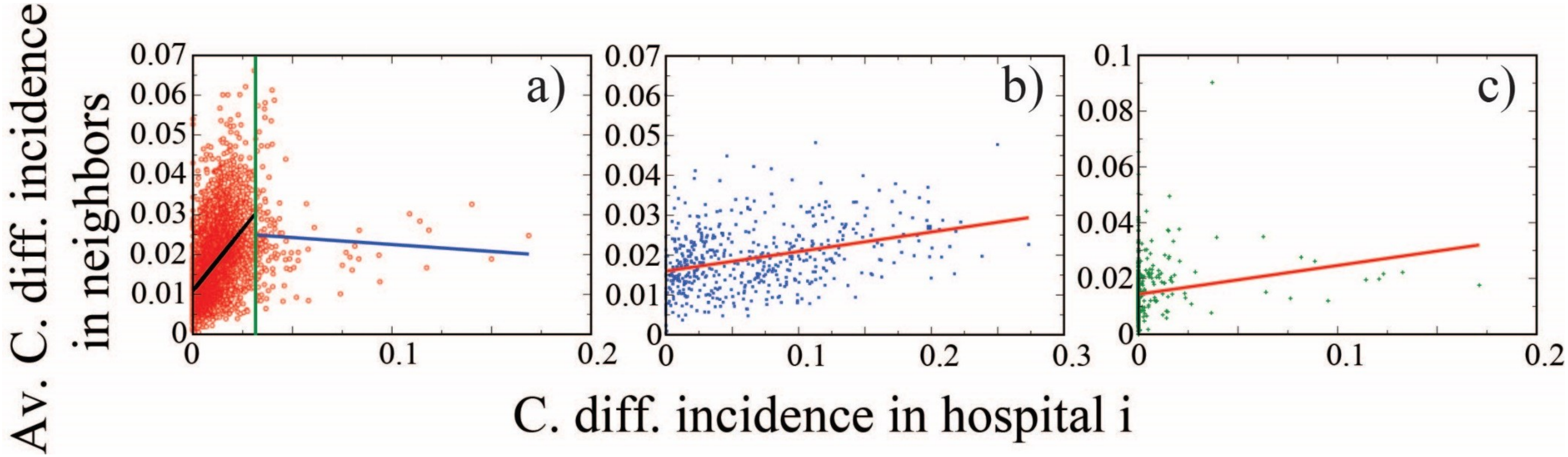

Fig. S4. ***C. difficile*** **incidence in a hospital and its neighbors.** Here we plot the mean *C. difficile* incidence of a hospital on the x-axis and the average mean *C. difficile* incidence of the neighboring hospital in the transfer network, where a neighboring hospital is one that either sends it patients to or receives its patients from the case hospital. **a)** For general hospitals, two regimes appear that are separated by the vertical line. The straight lines within each regime are regression lines. **b)** For rehabilitation hospitals, only one regime appears (regression line shown). **c)** The remaining hospitals also form just one regime (regression line shown).

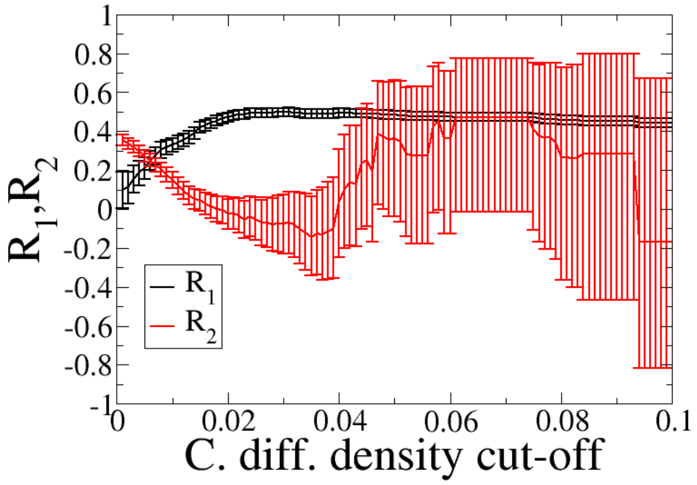

Fig S5. Correlation coefficients of the two regimes for mean *C. difficile* in general hospitals. The lines are Pearson correlation coefficients and the intervals around show the 95% confidence intervals. The black curve is for the correlation coefficient of the cloud in the low incidence regime, given the cut-off marked by the x-axis. The red one is for the high incidence regime.

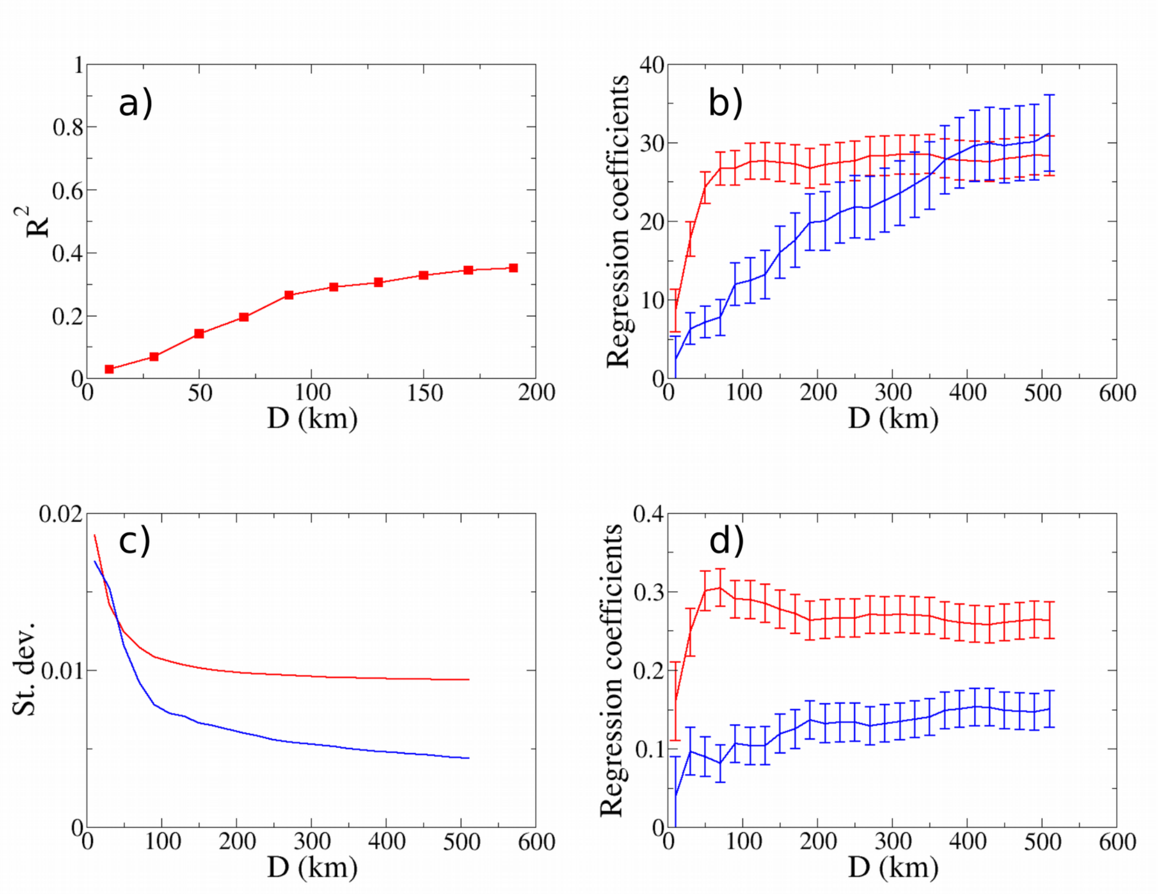

Fig.S6. Effect of geographical neighbors vs. network restricted geographical neighbors. a) Shows the squared Pearson correlation coefficient corresponding to the indicator variables, as a function of distance *D*. **b)** The x-axis shows the value of the distance d up to which we pick neighboring hospitals for the averages in the neighborhood (restricted to network neighbors, $\langle \rho \rangle_i^{Net}(D)$, or not network neighbors, $\langle \rho \rangle_i^{Com}(D)$). Coefficient β (see equation in "**Community acquired infections vs. hospital acquired infections")** is represented by the red curve and ɣ by the blue curve. Error bars show the 95% confidence intervals. We see that the effect of connected hospitals is bigger for connected hospitals than those which are not at least up to 250km. **c)** Standard deviation of the network indicators (red) and of the community indicator (blue) as a function of distance. **d)** Result of the regressions after standardization. The results support the results in plot b (same color codes).

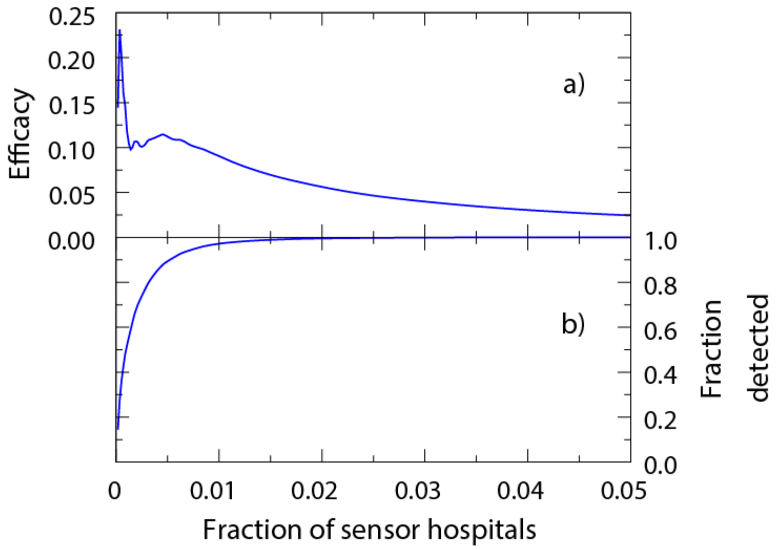

Fig S7. Finding the optimal number of sensors for the best sensor selection (static network). a) shows the efficacy and **b)** the fraction of detected cases, both as a function of the fraction of hospitals used as sensors. There is a peak for a very low fraction of sensors, but this point however corresponds to no more than 30% of detected cases. The second peak located at around 0.005 (using 0.5% of hospitals as sensors) is able to detect over 80% of the cases.

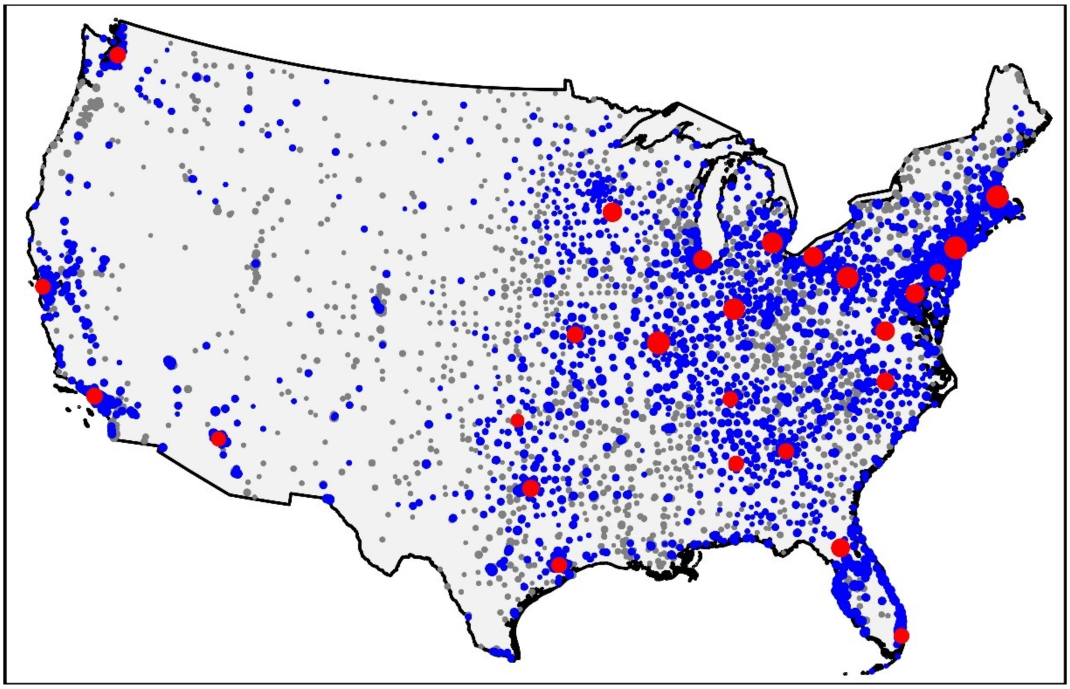

Fig. S8 Spatial positioning of the optimal sensor set. Red dots represent the sensor hospitals and blue dots are (nearest) neighbors of sensor hospitals. The size of each dot represents the mean C. diff incidence taken over the 2-year period at the hospital. The maps were created using the Basemap Matplotlib Toolkit 1.0.8 (http://matplotlib.org/basemap/) for Python [S6].

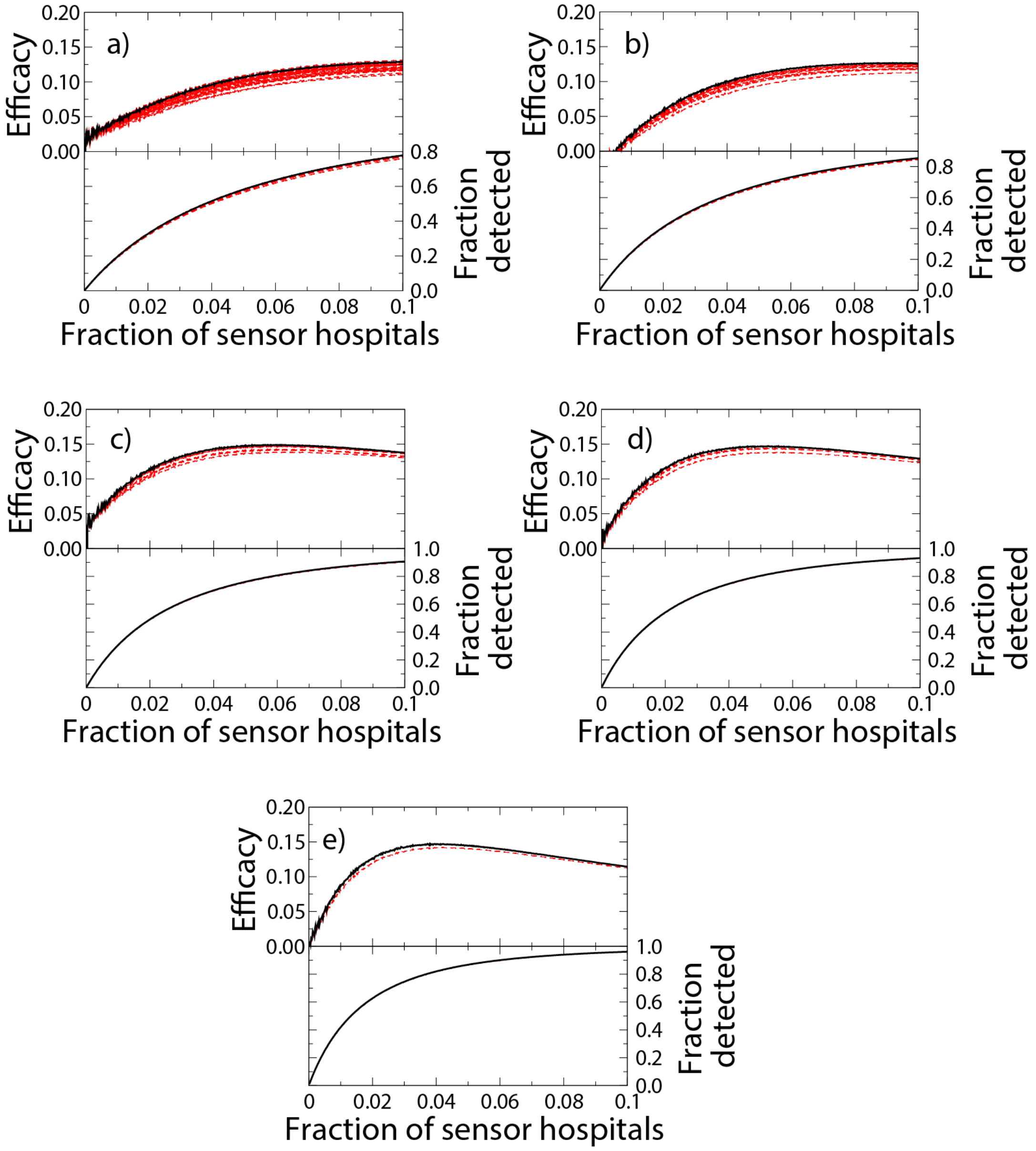

Fig. S9 Out-degree strategy: training vs. test data. Due to temporal correlations in the data, the sensor sets derived from the first slice of data perform comparably to their performance on the training set when applied to the remaining slices of data as test data. In all the plots, the results for the training set are shown as black solid lines while the red dashed lines refer to the sensor set applied to the test data sets. From left to right and top to bottom, the different plots refer to window widths of 1 (a), 2 (b), 4 (c), 6 (d), and 12 (e) months.

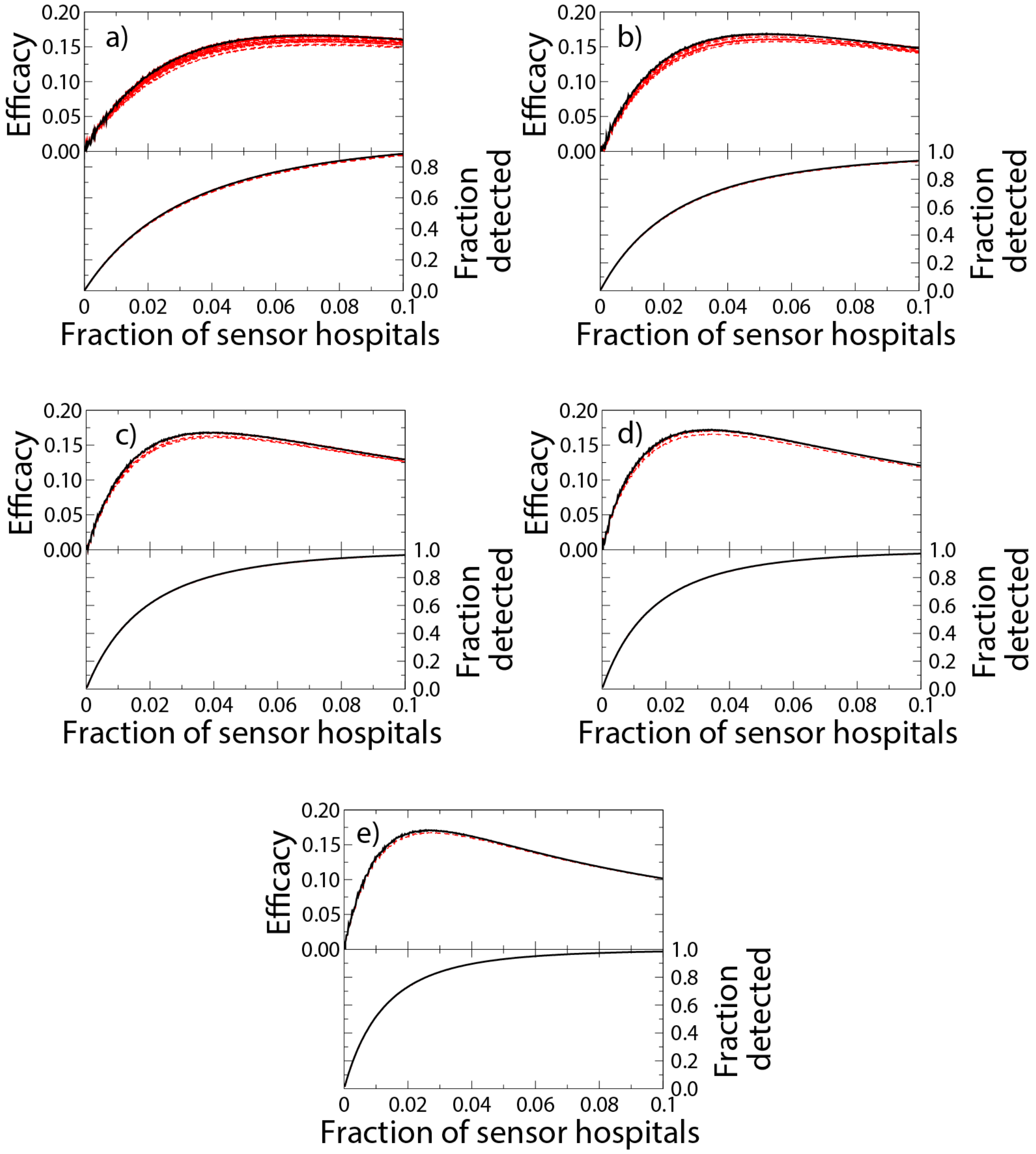

Fig. S10 In-degree strategy: information vs. validation sets. The panels are arranged as above. Due to the temporal correlation of the data the sensor sets derived from the first slice of data perform comparably to their performance on the training set when applied to the remaining slices of data as test data.

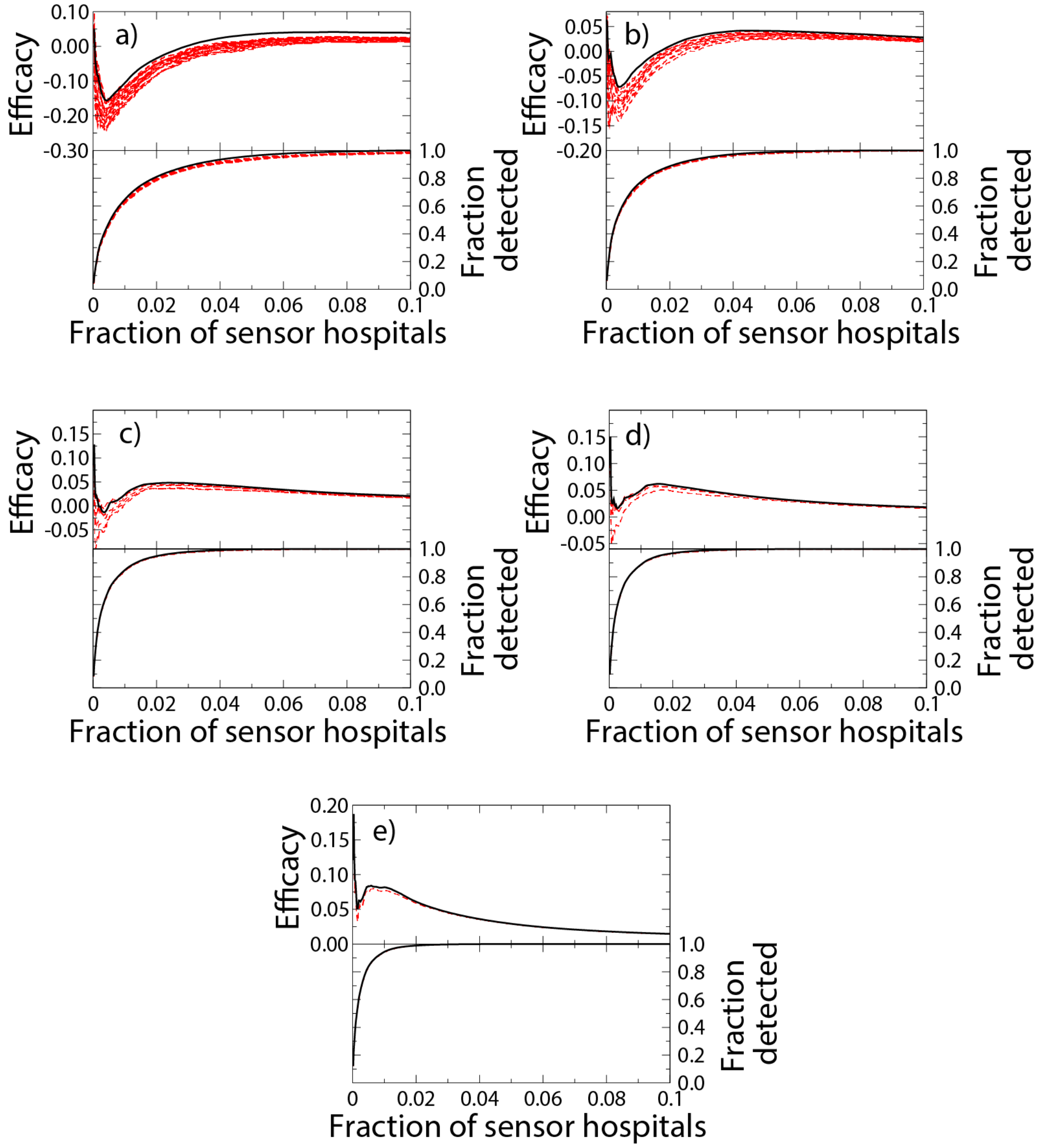

Fig. S11 Greedy strategy: information vs. validation sets. The panels and are arranged as above. Due to the temporal correlation of the data the sensor sets derived from the first slice of data perform comparably to their performance on the training set when applied to the remaining slices of data as test data. Nevertheless, when compared to the other strategies this is slightly more variable when compared training and test data results.

REFERENCES

S1. Newman MEJ, Strogatz SH, Watts DJ. (2001) Random graphs with arbitrary degree distributions and their applications. Phys. Rev. E 64(2):026118

S2. Newman M. (2002) Assortative Mixing in Networks. Phys. Rev. Lett. 89(20):208701

S3. Newman M. (2003) Mixing patterns in networks. Phys. Rev. E 67(2):026126

S4. Cormen, T.H., Leiserson, C.E., Rivest, R.L., Stein, C. (2001) Introduction To Algorithms, Chapter 16, MIT Press

S5. Kirkpatrick, S., Gelatt, C.D., and Vecchi, M.P. (1983) {\it Science} 220, pp. 671–680

S6. Hunter J.D. Matplotlib: A 2D graphics environment. *Computing in Science & Engineering,* **9**, 90-95 (2007).
